# Platelet-Derived Toll-Like Receptor 4 (Tlr-4) Is Sufficient to Promote Microvascular Thrombosis in Endotoxemia

**DOI:** 10.1371/journal.pone.0041254

**Published:** 2012-07-20

**Authors:** Ryan J. Stark, Niloufar Aghakasiri, Rolando E. Rumbaut

**Affiliations:** 1 Department of Pediatrics, Baylor College of Medicine, Houston, Texas, United States of America; 2 Department of Medicine, Baylor College of Medicine, Houston, Texas, United States of America; 3 Medical Care Line, Michael E. DeBakey Veterans Affairs Medical Center, Houston, Texas, United States of America; Charité, Campus Benjamin Franklin, Germany

## Abstract

Endotoxin (lipopolysaccharide, LPS) produced by gram-negative bacteria initiates a host of pro-inflammatory effects through Toll-like receptor 4 (TLR-4). We reported previously that LPS enhances microvascular thrombosis in cremaster venules of wild-type mice, but had no effect in mice deficient in TLR-4. Since TLR-4 is expressed on various cell types, the cellular origin of TLR-4 responsible for the LPS-enhanced thrombosis remains undetermined. Platelets are known to express functional TLR-4. Platelet-derived TLR-4 has been suggested to mediate various inflammatory responses in endotoxemia, including production of tumor necrosis factor alpha (TNF-α) and interleukin-1 beta (IL-1β), two cytokines reported to enhance microvascular thrombosis. We determined whether platelet-derived TLR-4 was sufficient to mediate the enhanced thrombosis induced by endotoxin and whether these responses were accompanied by systemic increases in TNF-α and IL-1β. We isolated platelets from wild-type mice and transfused them into either of two strains of TLR-4-deficient mice (C57BL/10ScN and B6.B10ScN-TLR-4^lps-del^/Jth). The mice were then injected with LPS or saline, and the kinetics of thrombosis were studied 4 hours later. Transfusion of wild-type platelets restored responsiveness to LPS in TLR-4-deficient mice with regards to microvascular thrombosis but not to plasma levels of TNF-α or IL-1β. The accelerated rates of microvascular thrombosis induced by platelet transfusions were specific to TLR-4, since isolation and transfusion of platelets derived from TLR-4-deficient donors did not restore responsiveness to LPS. These studies demonstrate that platelet-derived TLR-4 is sufficient to promote microvascular thrombosis in endotoxemia, independent of systemic increases in TNF-α or IL-1β.

## Introduction

Sepsis, defined as a systemic inflammatory response secondary to an infection, is a leading cause of mortality in populations worldwide [1]. One consequence of this syndrome is a systemic alteration in coagulation, called disseminated intravascular coagulation, which is associated with multi-organ system dysfunction and higher mortality [2], [3]. Many manifestations of sepsis, including a pro-thrombotic state, are reproduced by injection of endotoxin (lipopolysaccharide, LPS), a component of the cell wall of gram-negative bacteria [4], [5]. However, the mechanisms by which endotoxemia promotes thrombosis remain to be fully characterized.

A key molecule in signaling by LPS is Toll-like receptor (TLR)-4. TLRs are transmembrane proteins found on a multitude of mammalian cells and are involved in pathogen recognition and activation of the innate immune system [6]. While a number of TLRs have been described and our understanding of their individual roles in the inflammatory cascade is evolving, TLR-4 is critical for LPS-induced systemic responses [7], [8]. Previous data from our laboratory demonstrate that LPS promotes microvascular thrombosis in a photochemical injury model in mouse cremaster venules; this response was absent in mice deficient in TLR-4 [9]. TLR-4 can be found on a wide variety of cells including platelets [10], endothelial cells [11], and monocytes [12], however which cells expressing TLR-4 are responsible for the LPS-enhanced microvascular thrombosis remains unclear. While there has been evidence to support a functional role of TLR-4 on both platelets [10] and endothelial cells [11], there is increasing evidence supporting the role of platelets serving as an important link between the innate immune system and thrombosis.

Platelets are known to interact with leukocytes during sepsis [13] and inhibition of a platelet adhesion molecule, P-selectin, can prevent leukocyte rolling and adhesion [14]. Likewise, depletion of platelets can prevent neutrophil recruitment to sites of inoculation with LPS [15]. This role of platelets in innate immunity may come from the platelets themselves, as platelets contain RNA of pro-inflammatory cytokines, such as IL-1β, that can be translated and expressed by platelets exposed to LPS [16]. Cytokine expression via systemic inflammation may further be dependent on platelets, as levels of TNF-α, another pro-inflammatory cytokine, can be reduced by rendering mice thrombocytopenic and restored by transfusing them with platelets [17]. Further, both TNF-α and IL-1β have been suggested to mediate microvascular thrombosis in a photochemical-injury induced model in mice [18], [19]. Clearly, platelets play an important role in both thrombosis and inflammation, and the pro-inflammatory states induced by LPS may be through a platelet-derived TLR-4 mechanism. Given these findings, we hypothesized that platelet-derived TLR-4 was instrumental in the pro-thrombotic response to LPS, accompanied by systemic increases in circulating TNF-α and IL-1β.

## Materials and Methods

### Mice

We studied male mice between 25 to 30 g of weight. Strains included C57BL/6 and C57BL/10 (wild-type) and two strains of TLR-4-deficient mice. Both strains of TLR-4-deficient mice (C57BL/10ScN, and B6.B10ScN-TLR-4^lps-del^/Jth) were purchased as homozygous knockouts. All strains originated from the Jackson Laboratories (Bar Harbor, ME). All mice were studied in strict accordance with the recommendations in the Guide for the Care and Use of Laboratory Animals of the National Institutes of Health. Protocols used within this study were approved by the Animal Care and Use Committee of Baylor College of Medicine (Permit Number: AN-1996). All procedures were performed under sodium pentobarbital anesthesia as outlined below, and all efforts were made to minimize suffering.

### Animal Preparation

Mice were anesthetized with intraperitoneal (IP) injections of sodium pentobarbital (50 mg/kg), with additional doses (25 mg/kg) as needed. Mice were then placed on a custom Plexiglas tray and maintained at 37°C with a homeothermic blanket monitored through a rectal temperature probe (F.H.C.). A tracheotomy was performed to facilitate breathing and cannulation of the internal jugular vein and carotid artery were performed for intravenous drug administration and hemodynamic monitoring, respectively. The cremaster muscle was then exteriorized and prepared for intravital microscopy as described previously [9], [20].

### Platelet Isolation and Transfusion

To determine whether platelet-derived TLR-4 was sufficient to promote microvascular thrombosis *in vivo*, mice were anesthetized as described above and whole blood was collected from the carotid artery using a 1 mL syringe containing 0.1 mL Acid-Citrate-Dextrose solution (Sigma, MA). Platelet-rich plasma (PRP) was obtained from whole blood by centrifugation at 260×g for 8 min and 260×g for an additional 3 minutes, collecting supernatant after each centrifugation. The PRP was then spun at 740×g for 10 minutes and the supernatant was discarded. The pellet containing platelets was then re-suspended in 0.5 mL of Phosphate Buffered Saline (Sigma, MA) and allowed to sit for 30 minutes. Platelet concentration was determined by taking 10 µL of washed platelet suspension, diluting it using the Unopette collection system (Becton Dickinson, Franklin Lakes, NJ, 1∶100 dilution) and counting the platelets with a hemocytometer. As reported previously, this method of platelet isolation results in <0.01% of leukocytes in the platelet suspensions [9]. Platelets from suspension were then diluted with PBS to a concentration of 200×10^6^ platelets in 0.15 mL, and transfused via tail vein into recipient mice just prior to LPS or saline administration. This platelet dose corresponds to approximately 10% of the circulating platelets; a proportion of exogenous platelets shown to be sufficient to mediate several inflammatory responses in P-selectin-deficient mice [21], [22].

### Intravital Microscopy and Microvascular Thrombosis Model

Methods involving intravital microscopy and light/dye injury were performed as previously described [9], [20]. Briefly, prepared mice were placed under an upright microscope (BX-50, Olympus, NY) and observed with a 40× water immersion objective (N.A. 0.8). After an equilibration period of 30 minutes, fluorescein isothiocyanate (FITC)-dextran (150 kDa, 10 mL/kg of a 5% solution) was injected via the venous catheter and allowed to circulate for approximately 10 minutes. After this period, mean blood cell velocity (V_doppler_, using an optical Doppler velocimeter (Cardiovascular Research Institute, Texas A&M University) and venular diameter were measured (image 1.6, NIH, public domain software) to approximate the venular wall shear rate (γ) calculated as [8(V_doppler_/1.34)]/diameter [23].

After those measurements, light/dye-induced injury was initiated by exposing approximately 100 µm of a venule length to epi-illumination using a 175W xenon lamp (Lambda LS) and a fluorescein filter cube (HQ-FITC, Chroma). The excitation light was monitored daily (IL 1700 Radiometer, SED-033 detector, International Light) and maintained within a 1% of 0.6 W/cm^2^. Epi-illumination was applied continuously and the following times were recorded: (1) Time of onset of platelet aggregates and (2) Time of flow cessation, for a minimum of 60 seconds.

### Cytokine Analysis

Whole blood was collected 4 hours after platelet transfer and injection with either endotoxin or saline.

Plasma was isolated from whole blood by running blood samples at 1000×g for 10 minutes, collecting supernatant and freezing at −80°C until all samples were collected. Plasma samples were then thawed at room air and ELISA was performed for TNF-α (BD Biosciences, San Jose, CA) and IL-1β (R&D Systems, Minneapolis, MN) according to the manufacturer's instructions.

### Platelet Aggregation

Mice were anesthetized and PRP was collected as described above. Platelet concentration was determined by obtaining 5 µL of PRP, diluting the sample in the Unopette collection system (Becton Dickinson, Franklin Lakes, NJ, 1∶100 dilution) and counting platelets with a hemocytometer. PRP samples were then corrected to a concentration of 250×10^6^ platelets/mL with Tyrode's buffer (138 mM sodium chloride, 5.5 mM glucose, 12 mM sodium bicarbonate, 2.9 mM potassium chloride, and 0.36 mM sodium phosphate dibasic, pH 7.4). Platelet aggregation in response to various concentrations of ADP (Bio/Data Corporation, Horsham, PA) and collagen (Helena Laboratories, Beaumont, TX) indicated in the results section was measured in 225 µL of PRP at 37°C and at 1200 rpm in a four-channel Bio/Data PAP-4C aggregometer (Bio/Data Corporation, Horsham, PA).

### Experimental Groups

To determine the influence of endotoxemia on microvascular thrombosis, mice were injected intraperitoneally with either sterile saline or endotoxin from *Escherichia coli* serotype 0111:B4 (Sigma #L3024, endotoxin content 1×10^6^ EU/mg) at 5 mg/kg in 0.5 mL of sterile, pyrogen-free isotonic saline, 4 hours before photoactivation. For studies examining the effect of LPS on platelet aggregation, PRP was isolated as previously stated and samples were corrected to a concentration of 250×10^6^ platelets/mL with Tyrode's buffer. PRP was incubated with either LPS (18.75 µg/mL) or saline for 10 minutes prior to aggregation experiments. This LPS concentration is in the range of predicted maximal blood concentration, calculated from the intraperitoneal doses of LPS used for the *in vivo* experiments, and comparable to LPS concentrations (1–10 µg/mL) reported to enhance human platelet aggregation responses in similar experiments [24]. For all experiments, the investigator performing intravital microscopy or platelet aggregometry was blinded with regards to the injected agent (saline versus LPS) and the source of transfused platelets. At the conclusion of the intravital microscopy experiments, 10 µL of whole blood was collected via carotid artery cannulation, diluted with a Unopette collection system (Becton Dickinson, Franklin Lakes, NJ 1∶100 dilution) per the manufacturer's instructions, and platelets were counted with a hemocytometer.

### Statistics

Data are expressed as mean ± SD or SE, as specified. Comparisons of thrombosis times were done with two-tailed unpaired Student T-tests, aggregometry and cytokine level data were analyzed with one-way ANOVA, with Bonferroni correction for multiple-group comparisons by using GraphPad Prism 4.03 statistical software (GraphPad Software Inc., La Jolla, CA). A probability value of <0.05 was considered statistically significant.

## Results

### Mice

We performed intravital microscopy experiments in 141 mice with an average weight of 28.8 g (SD 2.6) and mean arterial pressure (MAP) of 84.7 mmHg (SD 11.1). There were no significant differences in weight or MAP between the groups. Microvascular thrombosis was induced in venules of average diameter of 45.2 µm (SD 2.3) and wall shear rate of 486 s^−1^ (SD 94); these parameters did not differ statistically between the groups.

### Platelet TLR-4 restored thrombotic responsiveness to LPS in TLR-4-deficient mice

**Figure 1:**
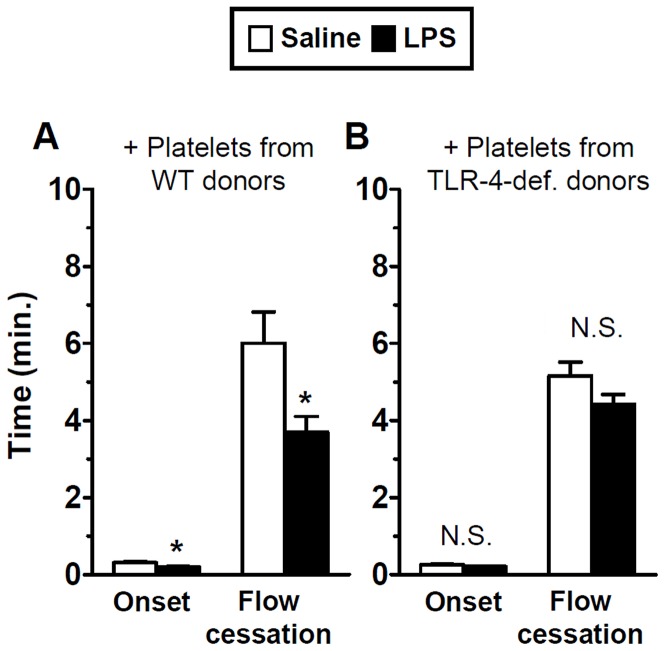
Platelets from wild-type mice restore LPS-enhanced thrombosis in TLR-4-deficient mice in a BL/10 background. TLR-4-deficient mice in a C57BL/10 background were transfused with platelets from either wild-type (A) or TLR-4-deficient (B) mice and exposed to saline or LPS. Microvascular thrombosis kinetics were assessed by a light-dye injury model using intravital microscopy. Initial platelet adhesion to the microvascular wall (Onset) and vessel occlusion for 60 seconds (Flow Cessation) were recorded in minutes. Data shown as mean ± SE, n = 12 per group, *:p<0.05 (for the comparison between saline and LPS by unpaired t-tests).

The influence of endotoxin exposure on microvascular thrombosis in a photochemical injury model following platelet transfusion into TLR-4-defcient mice (C57BL/10ScN strain) is shown in Figure 1. We reported previously that LPS had no effect on microvascular thrombosis in these mice, although it did enhance thrombosis in the corresponding wild-type control strain [9]. Following transfusion of platelets from wild-type donor mice (C57BL/10) into TLR-4-deficient mice, exposure to LPS enhanced the kinetics of microvascular thrombosis relative to saline (Figure 1A); both times to onset of thrombosis and flow cessation were significantly lower in LPS-treated mice, by approximately 40% (p<0.05). To exclude the possibility that the enhancement of thrombosis was a result of platelet activation during isolation and transfusion of platelets, we performed a control experiment by transfusing platelets derived from TLR-4-deficient donor mice into TLR-4-deficient recipient mice. As shown in Figure 1B, following transfusion of platelets from TLR-4-deficient mice, there was no significant difference in thrombosis times between Saline- or LPS-treated mice (n = 12 per group, N.S.).

**Figure 2:**
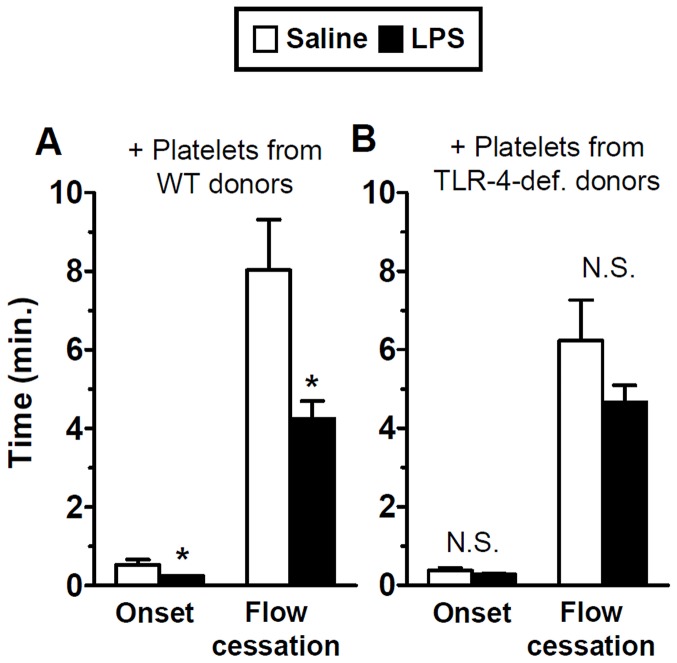
Platelets from wild-type mice restore LPS-enhanced thrombosis in TLR-4-deficient mice in a BL/6 background. TLR-4-deficient mice in a C57BL/6 background were transfused with platelets from either wild-type (A) or TLR-4-deficient (B) mice and exposed to saline or LPS. Microvascular thrombosis kinetics were assessed by a light-dye injury model using intravital microscopy. Initial platelet adhesion to the microvascular wall (Onset) and vessel occlusion for 60 seconds (Flow Cessation) were recorded in minutes. Data shown as mean ± SE, n = 12 per group, *:p<0.05 (for the comparison between saline and LPS by unpaired t-tests).

To confirm these findings in mice of a more commonly used genetic background (C57BL/6), we studied a second strain of TLR-4-deficient mice (B6.B10ScN-TLR-4^lps-del^/Jth). First, we assessed their responsiveness to LPS in the absence of platelet transfusion and confirmed our previously published findings in the BL/10 strain of TLR-4-deficient mice [9]; the kinetics of thrombosis did not differ between Saline- and LPS-treated groups (n = 12 per group, N.S., data not shown). Transfusion of platelets from wild-type (C57BL/6) donors into this second strain of TLR-4-deficient mice also restored their responsiveness to LPS with regards to microvascular thrombosis (Figure 2A); with findings comparable to those shown in the BL/10 strain (Figure 1A). Both the times of thrombosis onset and flow cessation were significantly lower in LPS-treated mice, by approximately 50% (p<0.05). As was the case in the BL/10 strain, the enhancement of thrombosis kinetics was not consequent to platelet activation during isolation and transfusion, since LPS had no significant effect on thrombosis kinetics after transfusion of platelets from TLR-4-deficient donors (n = 12 per group, N.S., Figure 2B).

### Platelet TLR-4 failed to restore cytokine responsiveness to LPS in TLR-4-deficient mice

**Figure 3:**
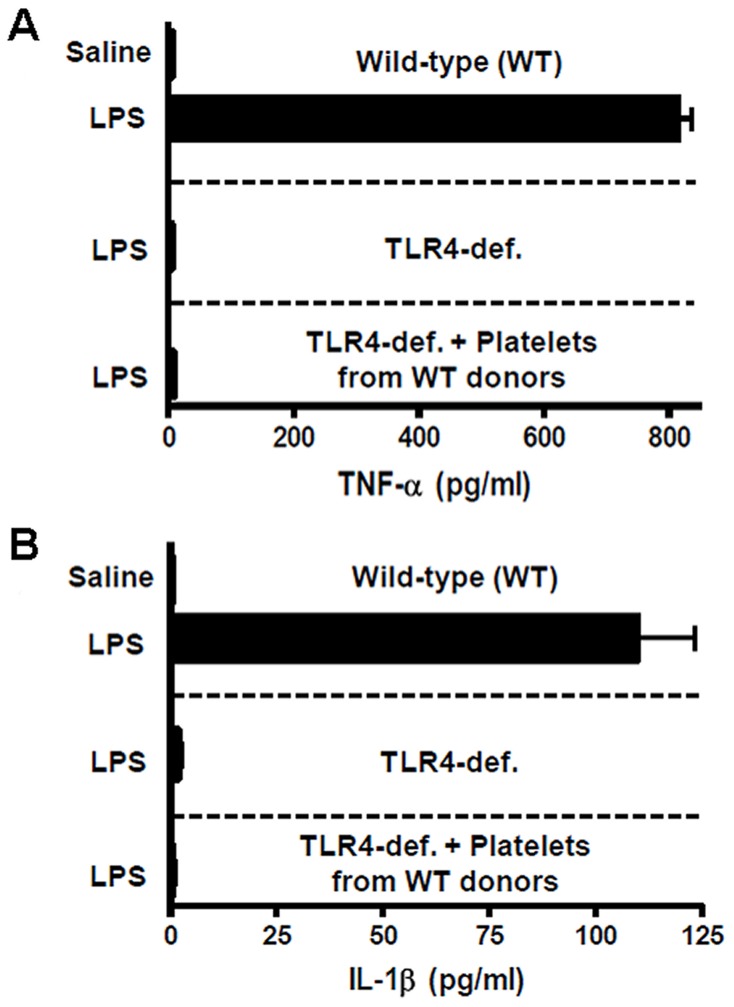
Platelets from wild-type mice do not restore LPS-enhanced cytokine release in TLR-4-deficient mice. Plasma cytokine levels of TNF-α (A) and IL-1β (B) were measured by ELISA in response to LPS in wild-type mice, TLR-4-deficient mice, and TLR-4-deficient mice transfused with platelets from wild-type mice. All TLR-4-deficient mice were of a C57BL/6 background. Data shown as mean ± SE, n = 4 per group. Analysis was done with one-way ANOVA with Bonferroni correction.

To determine whether platelet-derived TLR-4 was sufficient to induce systemic cytokine responses to LPS in TLR-4-deficient mice on the BL/6 background, we measured plasma TNF-α and IL-1β in mice transfused with platelets from wild-type donors. As shown in Figure 3, LPS induced a marked increase in plasma TNF-α and IL-1β in wild-type mice. However, this systemic cytokine response was absent in TLR-4-deficient mice, despite transfusion of platelets from wild-type mice, under the same conditions which enhanced thrombotic responses to LPS (Figure 2A). These findings demonstrate that the role of platelet-derived TLR-4 in promoting microvascular thrombosis in endotoxemia is not accompanied by a systemic pro-inflammatory cytokine response.

### Platelet TLR-4 Expression does not influence Platelet Aggregation

**Figure 4:**
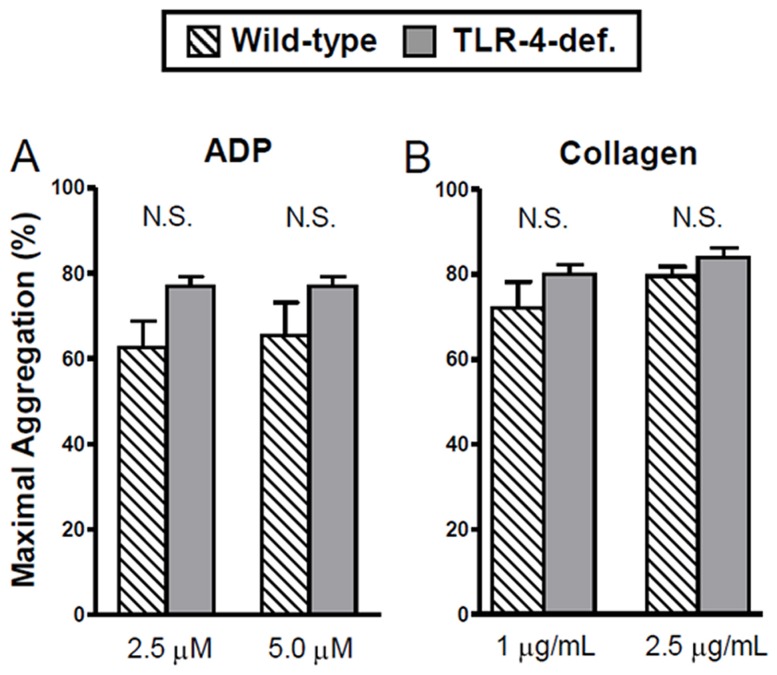
Platelet aggregation responses are comparable in wild-type and TLR-4-deficient mice. Maximal aggregation of platelet-rich plasma from TLR-4-deficient and wild-type mice in the presence of platelet agonist ADP (A) or collagen (B) across dose ranges. All TLR-4-deficient mice were of a C57BL/6 background. Data shown as mean ± SE, n = 4 to 8 per group. Analysis was done with one-way ANOVA with Bonferroni correction.

We determined whether platelet-derived TLR-4 promotes thrombosis by enhancing platelet aggregation. To address this, we obtained platelet-rich plasma (PRP) from both TLR-4 deficient mice on the BL/6 background and their wild-type counterparts, and stimulated them with either ADP (dose range 2.5 µM and 5.0 µM) or collagen (dose range 1.0 µg/mL and 2.5 µg/mL). As shown in Figure 4, TLR-4 expression on platelets did not enhance their aggregation; we found no significant differences in aggregation responses to ADP or collagen. To examine the effects of endotoxin on platelet aggregation, we exposed wild-type PRP to either saline or LPS and measured the aggregation response. Similar to the aggregation of the previous experiment, there was no difference in maximal aggregation between wild-type platelets exposed to LPS or saline over the same dose ranges of ADP and collagen (n = 4 to 6 per group, N.S., data not shown). Overall these experiments demonstrate that platelets from wild-type mice did not inherently aggregate more compared to TLR-4-deficient platelets and that LPS had no effect on the aggregation of wild-type platelets compared to those exposed to saline.

## Discussion

The primary finding of this study was that platelet-derived TLR-4 was sufficient to mediate enhanced microvascular thrombosis in endotoxemia. After transfusion of approximately 10% of circulating platelets from wild-type mice into TLR-4-deficient recipients, endotoxin accelerated the rate of microvascular thrombosis as compared to the respective saline controls (Figures 1A and 2A). This response was specific to TLR-4 and was not a result of platelet activation from isolation of platelets and transfusion, since isolation and transfusion of platelets from TLR-4-deficient donors failed to influence thrombotic responses to LPS (Figures 1B and 2B). The role of platelet-derived TLR-4 in these responses was comparable in both strains of TLR-4-deficient mice (C57BL/6 and C57BL/10 backgrounds). Interestingly, the pro-thrombotic response to LPS mediated by platelet-derived TLR-4 was not accompanied by a systemic increase in plasma cytokines levels. This dissociation between the pro-thrombotic and systemic pro-inflammatory cytokine responses in endotoxemia provides new insight into the role of platelet-derived TLR-4.

The mechanisms involved in the pro-thrombotic state in the microvasculature accompanying endotoxemia remain to be clarified. Studies on the effects of endotoxin on the microcirculation have emphasized endothelial activation and adhesion of blood cells, including platelets, to the activated endothelium [5]. Published reports differ on the relative contribution of platelets as primary mediators of microvascular inflammation in endotoxemia. For example, a report by Cerwinka et al. [25], suggested that endothelial cells, and not platelets, mediated platelet-endothelial adhesive interactions during endotoxemia. Similarly, endothelial cell TLR-4 was shown to be sufficient to clear lethal systemic gram-negative infections [26]. However, platelet-derived TLR-4 has been reported to mediate functional responses in endotoxemia, including platelet sequestration [10], thrombin generation [27], and both enhanced [24] and decreased activation of isolated platelets [28]. Our *in vivo* data expand significantly on these observations by demonstrating that platelet-derived TLR-4 is sufficient to promote a pro-thrombotic state in TLR-4-deficient mice following exposure to endotoxin (Figures 1 and 2).

We pursued a role for TNF-α and IL-1β based on published data linking these pro-inflammatory cytokines to platelet-dependent responses in endotoxemia and their reported role as mediators of microvascular thrombosis. For example, Aslam et al. [17] reported that platelets were necessary for LPS-induced production of TNF-α. Those authors showed that platelet depletion inhibited TNF-α production, and transfusion of platelets from wild-type mice into mice with defective TLR-4 signaling (C3H-deficient) was sufficient to induce increased serum TNF-α 1 hour following LPS. Similarly, endotoxin has been shown to induce splicing of platelet RNA in mice and accumulation of IL-1β protein [16]. Since TNF-α and IL-1β are known to promote microvascular thrombosis in a mouse model of photochemical injury [18], [19], systemic increases in these cytokines represented a likely mechanism to account for platelet TLR-4-dependent restoration of responsiveness to LPS with regards to thrombosis. However, as shown in Figure 3, despite transfusion of platelets from wild-type mice, TLR-4-deficient mice were unresponsive to LPS with regards to plasma cytokines. Our finding showing a lack of increase in TNF-α differ from those of Aslam et al, though methodological differences between the studies (differences in mouse strain, LPS preparation, time of plasma collection, etc.) preclude a direct comparison of the findings. Under our experimental conditions, these data demonstrate that platelet-derived TLR-4 can promote thrombosis independent of a systemic pro-inflammatory cytokine response. However, the lack of increased plasma levels of TNF-α and IL-1β do not entirely exclude an effect of these cytokines (i.e., locally) in the TLR-4-dependent enhancement of microvascular thrombosis.

The role of platelet-derived TLR-4 as a mediator of microvascular thrombosis in endotoxemia was unrelated to changes in platelet aggregation. As shown in Figure 4, *ex vivo* aggregation responses were comparable between wild-type and TLR-4-deficient mice. Moreover, LPS did not alter aggregation responses in platelets from wild-type mice, thus LPS-enhanced platelet aggregation does not appear to account for the pro-thrombotic responses to LPS. Our aggregometry findings are consistent with prior studies in murine endotoxemia [29], though some mixed results have been published [24]. However, our data suggest that platelet TLR-4 does not induce intrinsic differences in aggregation to account for its role in promoting microvascular thrombosis in endotoxemia.

Although we show that platelet TLR-4 promoted microvascular thrombosis without a systemic inflammatory cytokine response to endotoxin, our findings do not exclude a role for local microvascular inflammatory responses. For example, endotoxin was shown to promote release of IL-1β-rich microparticles from platelets [30]. Platelet-derived microparticles are associated with pro-thrombotic responses in macrovessels in a variety of models [31], [32], [33], and our previous data demonstrated that mice with impaired clearance of these microparticles have enhanced microvascular thrombosis in the same model used in this study [34]. Thus, it is conceivable that platelet-derived microparticles mediate platelet TLR-4-induced pro-thrombotic responses in endotoxemia. Other potential roles for platelet TLR-4 in microvascular responses include formation of neutrophil extracellular traps [35] and formation of platelet-leukocyte aggregates [36]. Although our studies demonstrate that platelet-derived TLR-4 was sufficient to restore LPS-enhanced thrombosis, it remains to be determined whether TLR-4-bearing platelets are exclusively responsible for this effect. Future studies are warranted to define a potential contribution of these mechanisms to the *in vivo* effects of platelet-derived TLR4 on enhanced microvascular thrombosis.

In conclusion, we demonstrate that platelet-derived TLR-4 is sufficient to mediate the enhanced microvascular thrombosis in endotoxemia independent of a systemic inflammatory cytokine response. These findings provide further evidence of a functional role of TLR-4 expression on platelets in endotoxemia and that a pro-thrombotic state can exist without evidence of increased systemic pro-inflammatory markers. The mechanisms by which TLR-4-mediated platelet responses interact with other components of the inflammatory response induced by endotoxin remain to be clarified.
